# Combined Metformin and Baricitinib Therapy Attenuates Inflammation in STZ‐Induced Diabetic Rats via AMPK/JAK–STAT Pathway Crosstalk

**DOI:** 10.1002/edm2.70101

**Published:** 2025-09-03

**Authors:** Mostafa Allahyari, AbdolJalal Marjani, Marie Saghaeian Jazi, Mehrdad Jahanshahi

**Affiliations:** ^1^ Metabolic Disorders Research Center Golestan University of Medical Sciences Gorgan Iran; ^2^ Department of Biochemistry and Biophysics Golestan University of Medical Sciences Gorgan Iran; ^3^ Neuroscience Research Center Golestan University of Medical Sciences Gorgan Iran

**Keywords:** AMPK, baricitinib, JAK–STAT, metformin, NF‐kB, SIRT1

## Abstract

**Background:**

Chronic inflammation is a critical factor contributing to diabetes complications. Baricitinib inhibits JAK–STAT signalling, which can contribute to an anti‐inflammatory effect. Similarly, metformin demonstrates anti‐inflammatory properties by activating the AMPK–SIRT pathway and suppressing the NF‐ᴋB signalling pathway. Here, we explored the effects of the coadministration of metformin and baricitinib in diabetic rats.

**Methods:**

Streptozotocin (40 mg/kg body weight) was administered to rats to develop diabetes after 2 weeks of 10% fructose solution consumption. The rats were treated with baricitinib (0.5, 2.5 and 5 mg/kg) and 150 mg/kg metformin for 1 month. A dose of 0.5 mg/kg baricitinib was chosen for combination therapy with metformin.

**Key Findings:**

Baricitinib induced significant weight loss at all three doses (*p* ≤ 0.05) and significantly increased lipid profile parameters in comparison to the diabetic control group (*p* ≤ 0.05). Pancreatic NF‐ᴋB levels and HOMA‐IR were meaningfully reduced in all treatment groups (*p* ≤ 0.01). Metformin and combination therapy significantly reduced serum TNF‐α levels (*p* ≤ 0.05). Furthermore, baricitinib at different doses and combination therapy significantly elevated serum IL‐10 levels (*p* ≤ 0.05). Additionally, combination therapy significantly upregulated the liver expression of NF‐ᴋB, SOCS1, SOCS3, AMPK and SIRT‐1 (*p* ≤ 0.01).

**Conclusion:**

Our results suggest that the coadministration of metformin with baricitinib reduces insulin resistance, improves histopathological alterations in the liver and pancreatic islet cells and counteracts the adverse effects of baricitinib on the lipid profile in diabetic rats. These findings hold particular significance for patients undergoing baricitinib treatment.

## Graphical abstract

Metaflammation is a key contributor to diabetes progression and its related complications. Coadministration of metformin with baricitinib mitigates metaflammation associated with diabetes and reduces insulin resistance. Graphical Abstract Created in BioRender.com.

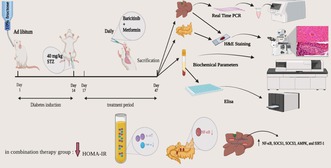

## 1 Introduction

Diabetes is a chronic, systemic and multifactorial disease caused by excessive blood glucose levels. It arises from inadequate insulin production by beta cells within the pancreas, tissues resistant to insulin, and an insufficient compensatory response in insulin production. In recent decades, diabetes prevalence has steadily increased around the world [1]. Complications such as nephropathy, retinopathy and neuropathy pose significant public health challenges, creating substantial social and economic burdens [2]. About 90%–95% of diabetes cases are type 2 [3]. Low‐grade inflammation has been linked to insulin resistance and type 2 diabetes in previous studies [4].

Diabetic patients newly diagnosed with type 2 diabetes are usually prescribed metformin (1,1‐dimethyl biguanide hydrochloride) as their first‐line treatment [5]. It has the potential to activate the 5′‐adenosine monophosphate‐activated protein kinase (AMPK) signalling pathway. By impeding the nuclear factor kappa‐light‐chain‐enhancer of activated B cells (NF‐ᴋB) signalling pathway, it can reduce the production of pro‐inflammatory cytokines. Additionally, clinical studies involving patients with impaired glucose tolerance indicate that treatment with metformin reduces the synthesis of pro‐inflammatory cytokines from inflammatory cells [6].

Cellular energy and homeostasis are regulated by AMP‐activated protein kinase (AMPK). It is stimulated in response to ATP depletion within cells and consequently increases the AMP to ATP ratio. AMPK enhances glucose uptake in muscle tissue and suppresses gluconeogenesis in the liver. Additionally, AMPK suppresses fatty acid synthesis while promoting fatty acid absorption and β‐oxidation, thereby improving the lipid profile. These characteristics establish AMPK as a master regulator of metabolism and an interesting therapeutic candidate for type 2 diabetes [7]. AMPK induces an anti‐inflammatory effect and mitigates oxidative stress by activating the SIRT‐1 signalling pathway [8].

Sirtuins are a family of histone and protein deacetylases specifically regulated by the NAD^+^/NADH ratio. They function as metabolic sensors and play a crucial role in linking cellular metabolic states to chromatin structure, which ultimately influences gene expression levels [9]. SIRT‐1 regulates numerous metabolic processes, such as lipid metabolism, homeostasis of glucose, insulin sensitivity and production [10].

Baricitinib is an adenosine triphosphate‐competitive kinase inhibitor that specifically binds to Janus Kinase 1 (JAK1) and Janus Kinase 2 (JAK2). In February 2017, it was approved for the treatment of mild to severe active rheumatoid arthritis. Baricitinib exhibits a nearly equal binding affinity for JAK1 (IC50 = 5.9 nmol/L) and JAK2 (IC50 = 5.7 nmol/L), while demonstrating minimal effect on JAK3 (IC50 > 400 nmol/L) [11].

Janus Kinase‐Signal Transducer and Activator of Transcription (JAK–STAT) signalling pathway describes primary intercellular signalling pathways that act as a downstream mediator of cytokines, hormones and various growth factors. A key role of JAK–STAT proteins is to regulate and maintain essential processes, such as inflammation [12]. Studies on knockout mice have highlighted the significant role of JAK signalling, particularly JAK2, in regulating metabolic activities, including glucose homeostasis, insulin resistance, obesity and energy expenditure [13].

JAK–STAT signalling pathway is regulated at multiple levels, including Suppressor of Cytokine Signalling (SOCS) proteins [14]. JAK–STAT and NF‐ᴋB signalling pathways are important in chronic inflammation because they elevate the production of pro‐inflammatory cytokines [15]. This, in turn, leads to the overexpression of SOCS proteins, which inhibit insulin signalling by preventing insulin receptor substrates (IRS) from binding to insulin receptors [16]. SOCS proteins, particularly SOCS1 and SOCS3, exert an inhibitory effect on insulin signalling, thereby linking cytokine signalling to insulin resistance. These proteins serve as the primary inhibitors of cytokine signalling by directly binding to cytokine receptors or JAK molecules [17]. JAK–STAT and NF‐ᴋB pathways are primarily synergistic in their interactions. NF‐ᴋB activates the JAK–STAT pathway by inducing the expression of cytokine genes. Additionally, activated STAT proteins, especially STAT3, can promote the sustained activation of NF‐ᴋB [18].

The interaction between the AMPK‐SIRT signalling pathway and the JAK–STAT pathway involves multiple mechanisms. Following the pharmacological activation of AMPK by metformin, it directly phosphorylates JAK1, thereby inhibiting the JAK–STAT pathway in various cell types [19]. On the other hand, NF‐ᴋB interacts with both signalling pathways. The activation of AMPK inhibits NF‐ᴋB through SIRT1. Conversely, the JAK–STAT pathway enhances NF‐ᴋB activation. Therefore, we hypothesise that there may be potential cross‐talk in which AMPK activation by metformin and JAK–STAT inhibition by baricitinib could serve as a promising target for type 2 diabetes treatment and its adverse effects.

## 2 Materials and Methods

### 2.1 Drugs and Chemical Substances

STZ was purchased from Sigma‐Aldrich (USA) and stored at −20°C. Metformin was provided by Minoo Pharmaceutical Company (Tehran, Iran). Baricitinib was obtained from Sobhan Oncology Pharmaceutical Company (Iran).

### 2.2 Animals

A total of 48 male Wistar rats were used in this study. The animals were 10 weeks old and weighed between 200 and 250 g. Rats were kept in polypropylene cages under controlled conditions (temperature of 25°C ± 2°C, humidity: 55% ± 5%). Their diet consisted of rat pellets, and they had free access to water. Rats were acclimated for 1 week before the experiments. Performing all animal procedures was in accordance with PREPARE guidelines [20], and the Golestan University of Medical Sciences Ethics Committee approved these procedures (approval ID: IR.GOUMS.AEC.1401.011).

### 2.3 Developing Type 2 Diabetes Mellitus

The animals were given 10% fructose water solution ad libitum for 14 days. After this period, streptozotocin (STZ) was dissolved in sodium citrate buffer (pH 4.5) and quickly injected intraperitoneally at 40 mg/kg body weight to induce diabetes. After 3 days, blood glucose concentrations were evaluated, and rats with blood glucose levels higher than 200 mg/dL were classified as diabetic [21].

### 2.4 Treatment Protocols and Animal Grouping

The dose of metformin was calculated based on the average dosage used in human clinical settings (500–2500 mg/day) and converted to the equivalent dose for rats [22]. For baricitinib dosing, a pilot study was conducted. An initial dose of 0.5 mg/kg baricitinib was calculated based on the guideline described by Nair AB et al. [22]. This initial dose was then multiplied by 5 and 10, resulting in doses of 2.5 and 5 mg/kg baricitinib, respectively.

There were eight groups of animals (*n* = 6 per group). Group I, the normal control, received regular water without streptozotocin (STZ) injection. Diabetes was developed in groups II–VIII. Group II, the diabetic control, received saline only. Group III was treated with the solvent of baricitinib (10% DMSO). Group IV was given metformin at a dose of 150 mg/kg/day [22]. Group V was treated with 0.5 mg/kg/day baricitinib and 150 mg/kg/day metformin. Group VI received 0.5 mg/kg/day baricitinib. Group VII received 2.5 mg/kg/day baricitinib. Group VIII received 5 mg/kg/day baricitinib. All treatment groups received oral (P.O.) administration once daily for 30 consecutive days.

After the treatment period finished, chloroform was used to anaesthetise the rats, and blood was obtained via cardiac puncture into gel‐containing tubes for serum separation. Pancreas and liver tissues were dissected immediately after euthanasia. After washing the tissues with saline, they were stored at –80°C for subsequent analysis.

### 2.5 Biochemical Parameters Measurement

Serum levels of fasting blood glucose (FBG), total cholesterol (TC), triglycerides (TG), high‐density lipoprotein (HDL) cholesterol and low‐density lipoprotein (LDL) cholesterol were evaluated using commercial kits following the manufacturer's procedures. Analyses were performed using a BS 380 Mindray Biochemistry Analyser. Serum concentrations of cytokines (IL‐10 and TNF‐α) were measured using commercial ELISA kits (Karmania Pars Gene, Iran). Insulin and SIRT‐1 serum concentrations were quantified using commercial ELISA kits (Fine Test, China). The homeostasis model assessment of insulin resistance (HOMA‐IR) was determined by the equation: [insulin (μIU/ml) × glucose (mmol/L)]/22.5. Additionally, the homeostasis model assessment of β‐cell function (HOMA‐β), an index of insulin secretion from pancreatic β cells, was determined by the equation: [insulin (μIU/mL) × 20]/[glucose (mmol/L) − 3.5] [23]. The conversion factor for insulin units was 1 μIU/mL = 6 pmol/L [24].

Pancreatic tissues were homogenised in a mild RIPA buffer containing 1 mM PMSF as a protease inhibitor. Based on the Bradford assay (Bio‐Rad), the total protein concentration was determined, and the standard curve was plotted with bovine serum albumin. NF‐kB levels in the tissue homogenates were quantified using the NF‐kB p105 subunit ELISA kit (Fine Test, China) according to the instructions provided by the manufacturer.

### 2.6 Real‐Time PCR

Extracting total RNA from tissues was performed using Trizol reagent (Behprep, Shiraz, Iran). Complementary DNA (cDNA) was synthesised using Parstous Ultra‐TM kit (Mashhad, Iran) following DNase I digestion (ATR‐MED, Iran). Real‐time PCR was run using the Step One Plus system (ABI, USA) with SYBR Green master mix containing high ROX (Ampliqon, Denmark). Table 1 presents the primer sequences used for amplifying target genes. Here are the thermal cycling parameters: 95°C for 15 min, followed by 40 cycles of 95°C for 22 s, 61°C for 30 s and 72°C for 40 s. As an internal control, gene expression levels were normalised to GAPDH. Calculation of fold changes was performed using the 2^−ΔΔCT^ method, normalised to the diabetic control group.

**TABLE 1:** Primer sequences.

GAPDH	AGGTTGTCTCCTGTGACTTC
	CTGTTGCTGTAGCCATATTC
AMPK	TTGCGTGTGCGAAGGAAGAACC
	GGAGTAGCAGTCCCTGATTTGGC
SOCS1	AGTGGGTGTGGAGGGTGAGATG
	ATGGAGAGGTAGGCGTGGAGT
SOCS3	CCCTTCCTTTTCTTTACCACCGA
	ACCGTTGACAGTCTTCCGACA
NF‐ᴋB	GTATGGCTTCCCGCACTATGG
	TCGTCACTCTTGGCACAATCTC
SIRT‐1	CGCCTTATCCTCTAGTTCCTGTG
	CGGTCTGTCAGCATCATCTTCC

### 2.7 Histopathological Study

The excised pancreas and liver were fixed in 10% buffered formalin for 48–72 h after washing with saline. Sections of 5‐μm thickness were cut using a microtome from paraffin blocks for morphological analysis. Following standard protocols, the sections were transferred to slides, deparaffinised with xylene and stained with haematoxylin and eosin (H&E). Five sections per animal and five random fields per section were examined blindly using a light microscope (Olympus DP73, Japan).

### 2.8 Statistical Analysis

To analyse data, GraphPad Prism 8 software (USA) was used. One‐way ANOVA or Kruskal–Wallis was used to compare groups, followed by Tukey's honest significant difference (HSD) test and Dunn's multiple comparison test. In the text and figures, results are expressed as mean ± standard error of the mean (SEM). *p* value < 0.05 was defined as statistical significance.

## 3 Results

### 3.1 Weight Changes

**FIGURE 1:**
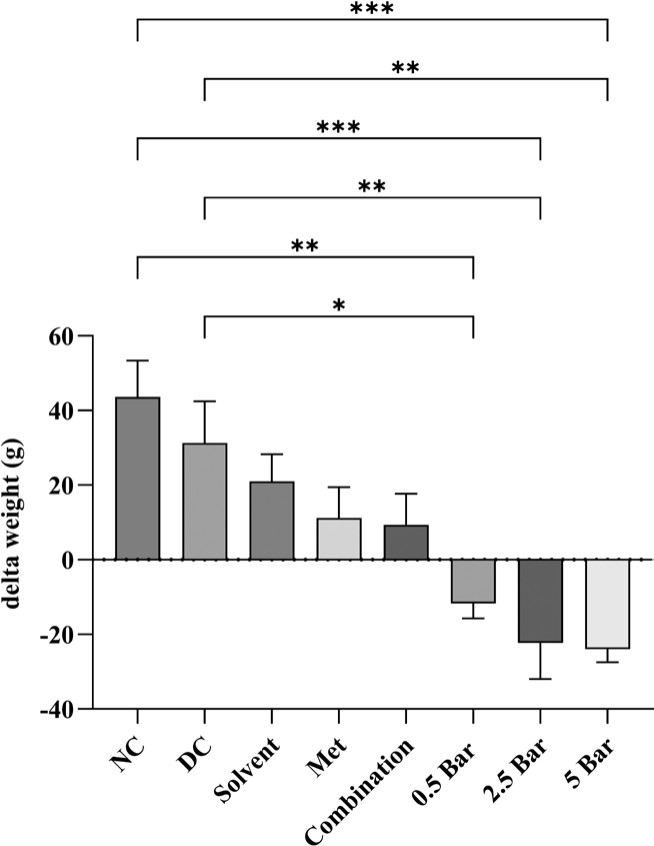
Body weight changes were monitored in the normal control (NC), diabetic control (DC) and treatment groups. Rats were given fructose for 2 weeks and then received a single injection of streptozotocin (STZ) to induce type 2 diabetes. The diabetic rats were subsequently treated for 1 month with 150 mg/kg of metformin, Baricitinib (0.5, 2.5 and 5 mg/kg), or a combination of metformin and 0.5 mg/kg of Baricitinib. *p ≤ 0.05, **p ≤ 0.01, ***p ≤ 0.001. Data are expressed as mean ± SEM.

Throughout the 4‐week treatment period, rats’ initial and final body weights were measured. Figure 1 shows the delta weight (final weight − initial weight). Treatment with baricitinib reduced delta weight significantly at doses of 0.5 mg/kg (−11.75 ± 3.9 g), 2.5 mg/kg (−22.3 ± 9.6 g) and 5 mg/kg (−24 ± 3.5 g) compared to the diabetic control group (31.2 ± 11.2 g) and to the normal control group (43.6 ± 9.8 g).

### 3.2 Biochemical Parameters

The effects of various treatments on biochemical parameters are presented in Table 2. In the diabetic control and solvent groups, FBG levels were significantly increased (*p* ≤ 0.05) compared to the normal control group. A significant difference could not be detected between all treatment groups and the diabetic control and normal control groups. Additionally, baricitinib treatment at 0.5 and 2.5 mg/kg meaningfully increased TC levels compared to the diabetic and normal control groups (*p* ≤ 0.05). Doses of 0.5, 2.5 and 5 mg/kg baricitinib significantly elevated TG levels compared to the diabetic control, normal control and combination treatment groups (*p* ≤ 0.0001). Furthermore, LDL‐C levels were significantly increased following 0.5 and 2.5 mg/kg baricitinib treatment in comparison to the diabetic control, normal control and combination treatment groups (*p* ≤ 0.05). HDL‐C levels increased significantly in 0.5 mg/kg baricitinib treatment groups compared to the diabetic control, normal control and combination treatment groups (*p* ≤ 0.05) (Table 2).

**TABLE 2:** Effects of different treatments on biochemical parameters.

Variables	NC	DC	Solvent	Met	Combination	0.5 Bar	2.5 Bar	5 Bar
FBG (mg/dL)	110.8 ± 19.59	295 ± 42.03^##^	228 ± 5.4^#^	166.2 ± 14.28	185.4 ± 7.99	159 ± 7	174 ± 1.73	174.3 ± 25.93
TG (mg/dL)	28.18 ± 3.8	32.83 ± 3.74	36.25 ± 1.97	30.56 ± 2.02	33.56 ± 5.87	78.5 ± 5.72^####^,****^,††††^	81.33 ± 5.04^####^,****^,††††^	83.25 ± 5.33^####^,****^,††††^
TC (mg/dL)	50.2 ± 4.4	50 ± 5.2	48.25 ± 3.8	55.6 ± 7.36	58.2 ± 7.66	85.5 ± 4.63^#^,*	86.33 ± 13.28^#^,*	71.5 ± 5.78
LDL‐C (mg/dL)	7.4 ± 0.81	7.5 ± 1.26	7.5 ± 1.55	7.40 ± 0.81	8 ± 0.71	14.75 ± 1.03^##^,**,^††^	14.33 ± 2.6^##^,*,^†^	11.75 ± 0.85
HDL‐C (mg/dL)	40.8 ± 5.36	40.5 ± 4.17	37 ± 2.68	44.4 ± 6.36	46.2 ± 6.38	75 ± 4.7^##^,*,^†^	68.67 ± 10.33	58.75 ± 7.2

*Note:* Met: 150 mg/kg/day metformin, Combination: Met + 0.5 mg/kg/day baricitinib, 0.5 Bar: 0.5 mg/kg/day baricitinib, 2.5 Bar: 2.5 mg/kg/day baricitinib, 5 Bar: 5 mg/kg/day baricitinib. *p ≤ 0.05, **p ≤ 0.01, ***p ≤ 0.001, ****p ≤ 0.0001 versus diabetic control group. # versus normal control group. † Versus combination group. Data are expressed as mean ± SEM.

Abbreviations: DC, diabetic control; FBG, fasting blood glucose; HDL‐C, high‐density lipoprotein cholesterol; LDL‐C, low‐density lipoprotein cholesterol NC, normal control; TC total cholesterol; TG, triglyceride.

Table 3 shows serum insulin levels and insulin sensitivity indices for various treatments. Insulin levels were not significantly different between the control groups and treatment groups. However, treatment with metformin, baricitinib (0.5, 2.5 and 5 mg/kg), and combination therapy significantly reduced the HOMA‐AIR index compared to the diabetic control group (*p* ≤ 0.01). Additionally, the HOMA‐B index displayed a meaningful increase in the 0.5 mg/kg baricitinib group compared to the diabetic control group (*p* ≤ 0.01).

**TABLE 3:** Effects of different treatments on serum insulin levels and insulin sensitivity indices.

Variables	NC	DC	Solvent	Met	Combination	0.5 Bar	2.5 Bar	5 Bar
Insulin (pg/mL)	151.5 ± 5.13	192.6 ± 12.8	185.4 ± 10.1	166.7 ± 12.39	176.9 ± 19.94	197.5 ± 11.1	179.7 ± 5.35	180.7 ± 19.98
HOMA‐IR	1.35 ± 0.23	4.3 ± 0.58^####^	3 ± 0.23^#^	1.98 ± 0.28***	2.2 ± 0.3**	2.23 ± 0.2**	2.2 ± 0.09**	2.33 ± 0.42**
HOMA‐B	23.7 ± 3.2	9.03 ± 0.85^##^	11.6 ± 0.28	18 ± 2.6	16.3 ± 1.4	21.5 ± 1.3**	16.75 ± 0.24	18.15 ± 1.9

*Note:* Met: 150 mg/kg/day metformin, Combination: Met + 0.5 mg/kg/day baricitinib, 0.5 Bar: 0.5 mg/kg/day baricitinib, 2.5 Bar: 2.5 mg/kg/day baricitinib, 5 Bar: 5 mg/kg/day baricitinib. *p ≤ 0.05, **p ≤ 0.01, ***p ≤ 0.001, ****p ≤ 0.0001 versus diabetic control group. # versus normal control group. Data are expressed as mean ± SEM.

Abbreviation: DC, diabetic control.

### 3.3 Serum Cytokines and Pancreatic NF‐ᴋB Levels

**FIGURE 2:**
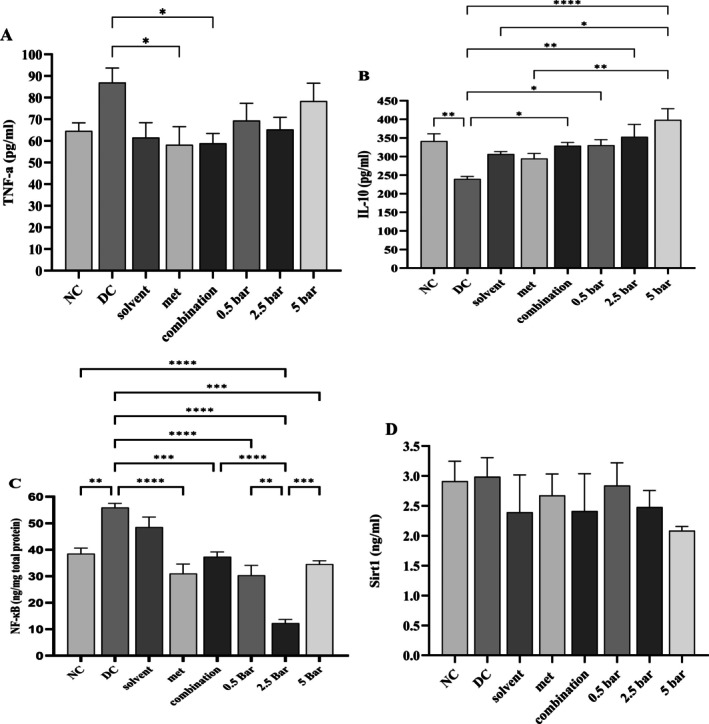
Serum levels of TNF‐α (A), IL‐10 (B), SIRT1 (D) and pancreatic NF‐κB (C) were analysed in the normal control (NC), diabetic control (DC) and treatment groups. Rats were given fructose for 2 weeks and then received a single injection of streptozotocin (STZ) to induce type 2 diabetes. The diabetic rats were subsequently treated for 1 month with 150 mg/kg of metformin, baricitinib (0.5, 2.5 and 5 mg/kg), or a combination of metformin and 0.5 mg/kg of baricitinib. *p ≤ 0.05, **p ≤ 0.01, ***p ≤ 0.001, ****p ≤ 0.0001 in comparison to the diabetic control group. Data are expressed as mean ± SEM.

Figure 2 illustrates the effects of different treatments on serum levels of TNF‐α, IL‐10, SIRT‐1 and pancreatic NF‐ᴋB.

#### 3.3.1 Serum TNF‐α

TNF‐α levels were significantly reduced in the metformin (58.18 ± 8.43 pg/mL) and combination (58.91 ± 4.49 pg/mL) therapy groups compared to the diabetic control group (86.98 ± 6.715 pg/mL). However, there was no significant difference between the other groups and the diabetic control group (Figure 2A).

#### 3.3.2 Serum IL‐10

IL‐10 levels in the diabetic control group were significantly lower (239.6 ± 6.89 pg/mL) than in the normal control group (341.5 ± 19.73 pg/mL). Baricitinib administration at 0.5, 2.5 and 5 mg/kg significantly increased serum IL‐10 levels (330.5 ± 14.68, 353 ± 33.5 and 398.6 ± 29.9 pg/mL, respectively) in comparison to the diabetic control group. Similarly, combination therapy (328.6 ± 9.3 pg/mL) also demonstrated a significant increase relative to the diabetic control group (Figure 2B).

#### 3.3.3 Pancreatic NF‐ᴋB

NF‐ᴋB levels in the normal control group were significantly lower (38.5 ± 2.1 ng/mg total protein) than in the diabetic control group (55.9 ± 1.58 ng/mg total protein). Baricitinib administration at 0.5, 2.5 and 5 mg/kg significantly reduced pancreatic NF‐ᴋB levels (30.37 ± 3.7, 12.29 ± 1.37 and 34.56 ± 1.3 ng/mg total protein, respectively). Similarly, treatment with metformin and combination therapy (31 ± 3.58 and 37.3 ± 1.8 ng/mg total protein, respectively) significantly decreased pancreatic NF‐ᴋB levels in comparison to the diabetic control group. Furthermore, pancreatic NF‐ᴋB levels showed a significant reduction in the 2.5 mg/kg baricitinib group compared to the 0.5 mg/kg and 5 mg/kg baricitinib and combination treatment groups (Figure 2C).

#### 3.3.4 Serum SIRT‐1

There were no significant differences between the treated, diabetic and normal control groups in serum SIRT‐1 concentrations (Figure 2D).

### 3.4 Histopathological Findings

#### 3.4.1 Liver

**FIGURE 3:**
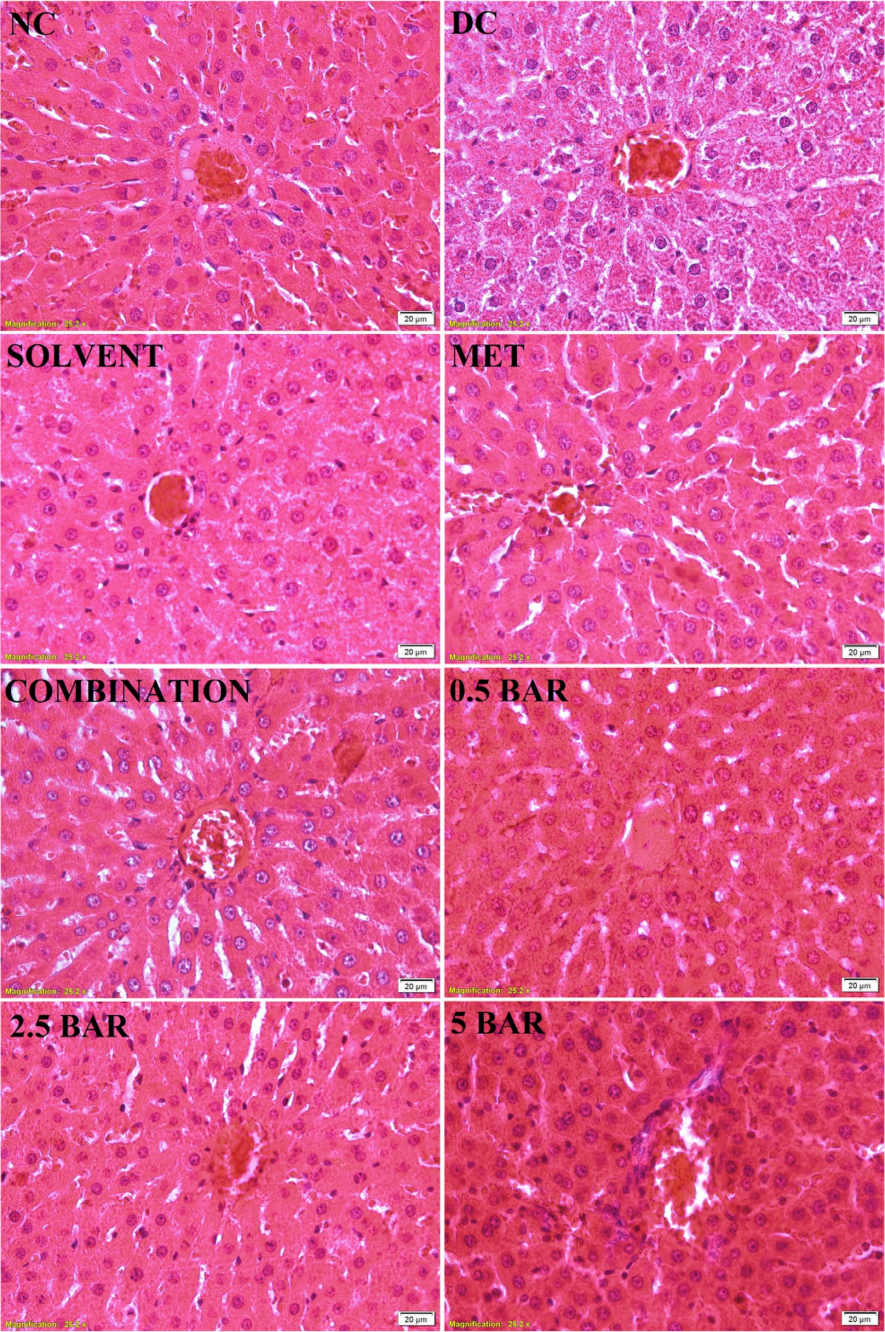
Photomicrographs of rat liver tissue (400× magnification) stained with haematoxylin and eosin (H&E) from control and treatment groups. Normal control (NC) rats are compared with SZT‐induced diabetic rats, which were divided into the following categories: Diabetic control (DC), 150 mg/kg Metformin (MET), combination (Metformin +0.5 mg/kg baricitinib), 0.5 BAR (0.5 mg/kg baricitinib), 2.5 BAR (2.5 mg/kg baricitinib) and 5 BAR (5 mg/kg baricitinib).

As illustrated in Figure 3, the normal control group exhibited typical hepatic cell architecture, characterised by organised cords radiating from the central vein. However, the liver of diabetic control rats exhibited dilation of sinusoids, hydropic degeneration, vacuolation of hepatocytes, disruption of hepatic cords and distortion of hepatic architecture relative to the normal control group. These pathological alterations were alleviated in various treatment groups, which demonstrate significant improvements in hepatocyte structure and architecture.

#### 3.4.2 Pancreas

**FIGURE 4:**
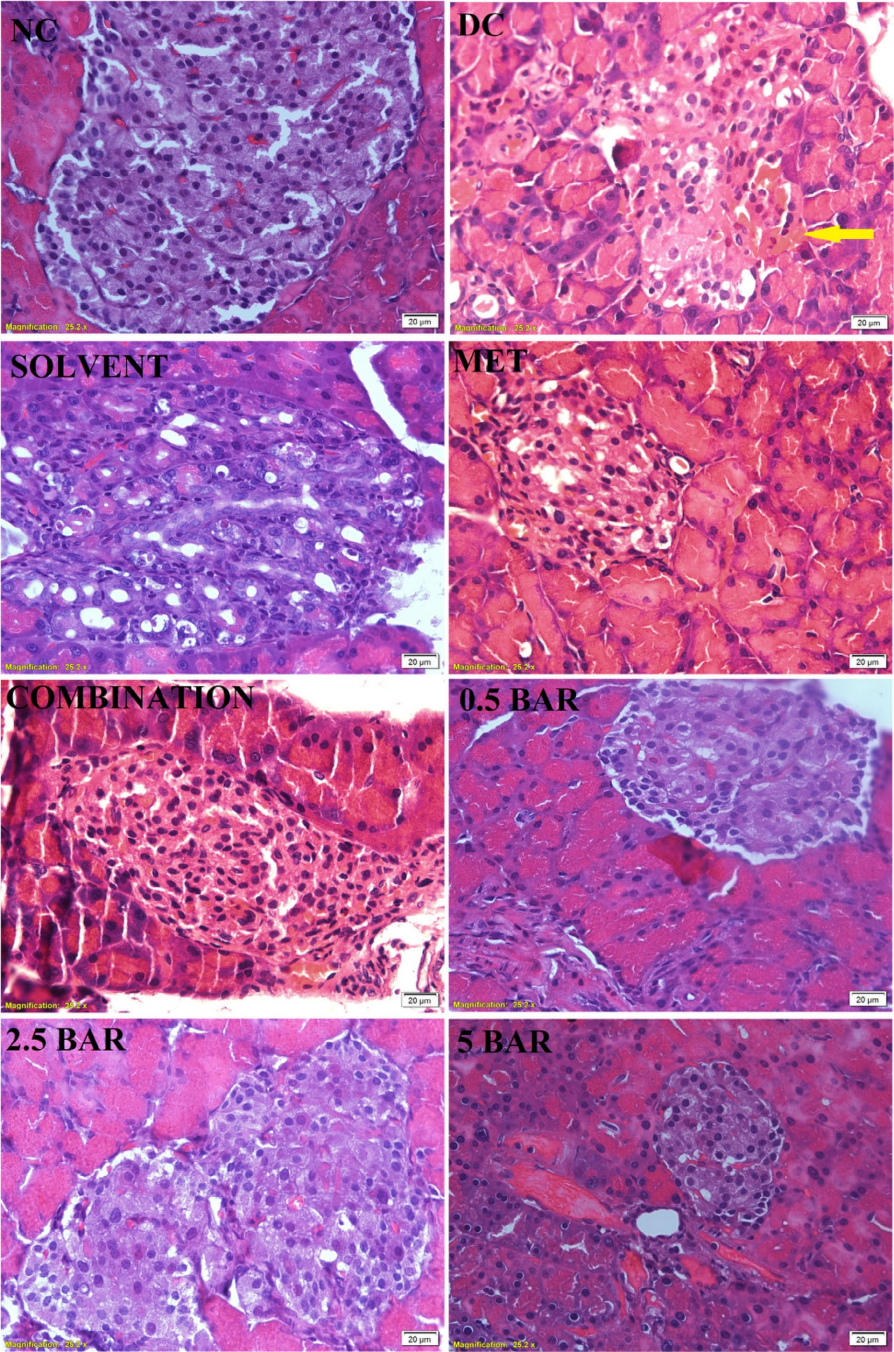
Photomicrographs of rat pancreatic tissue (400× magnification) stained with haematoxylin and eosin (H&E) from control and treatment groups. Normal control (NC) rats are compared with SZT‐induced diabetic rats, which were divided into the following categories: Diabetic control (DC), 150 mg/kg Metformin (MET), combination (Metformin +0.5 mg/kg baricitinib), 0.5 BAR (0.5 mg/kg baricitinib), 2.5 BAR (2.5 mg/kg baricitinib) and 5 BAR (5 mg/kg baricitinib).

As illustrated in Figure 4, pancreatic tissue from the normal control group exhibited a typical structure characterised by a high density of α cells and β cells, distinct tissue borders and well‐organised Langerhans islets. In contrast, the diabetic control and solvent groups displayed significant disruption of tissue architecture, including degeneration, shrinkage of the Langerhans islets, irregular borders, reduced density of α cells and β cells and increased haemorrhage in the surrounding area of the islet (indicated by yellow arrow). The various treatment groups demonstrated an increased number of cells within the islets, efficient regeneration and notable improvement compared to the diabetic control groups.

### 3.5 Gene Expression Findings

**FIGURE 5:**
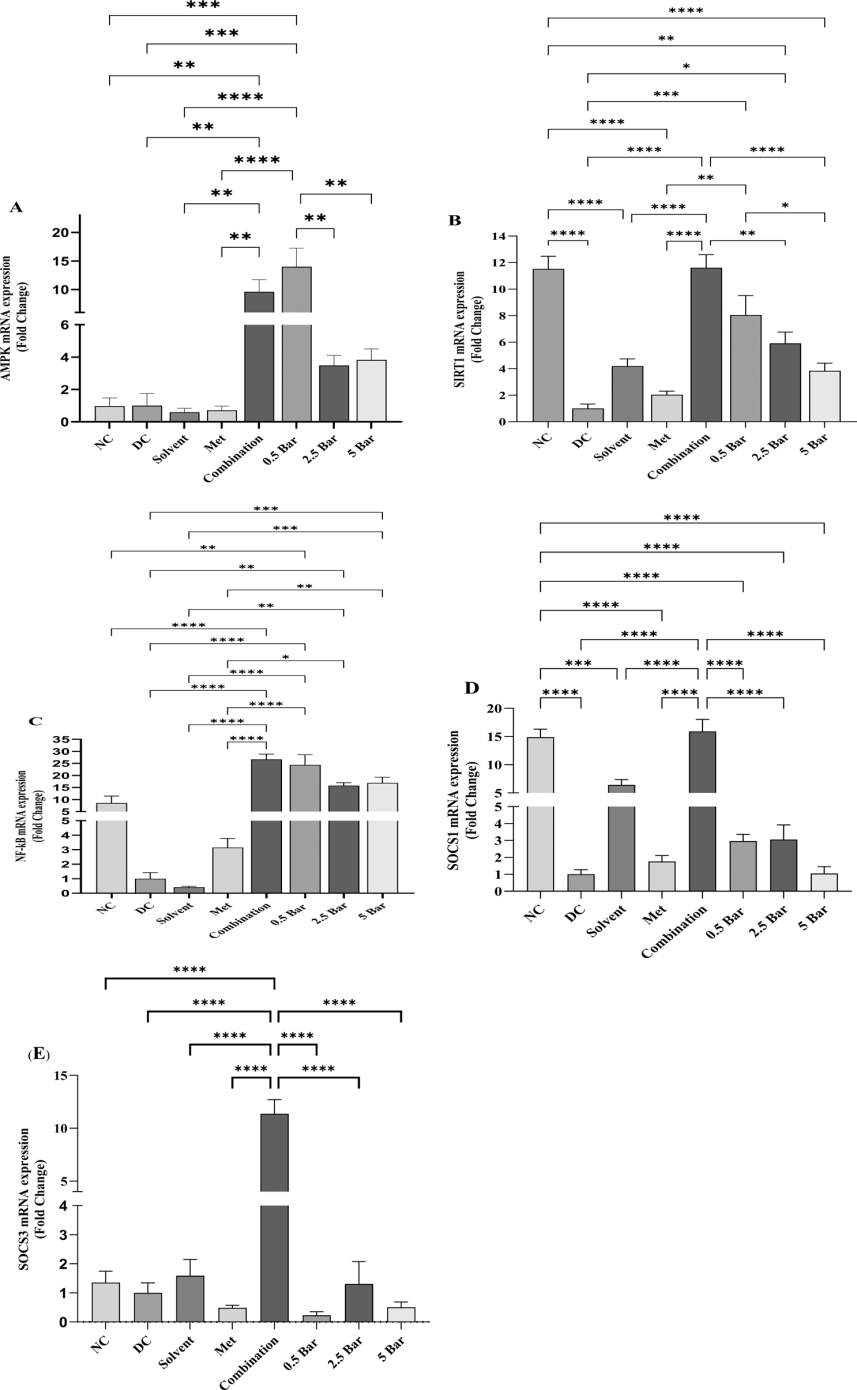
AMPK (A), SIRT1 (B), NF‐κB (C), SOCS1 (D) and SOCS3 (E), liver gene expression after receiving different treatments. Data are presented as mean ± SEM. Following is an indication of statistical significance: *p ≤ 0.05, **p ≤ 0.01, ***p ≤ 0.001 and ****p ≤ 0.0001.

The results of various treatments on liver gene expression of AMPK, SIRT1, NF‐ᴋB, SOCS1 and SOCS3 are illustrated in Figure 5.

#### 3.5.1 AMPK

Liver AMPK gene expression levels were significantly elevated in both the combination group [Fold Change (FC) = 9.6 ± 2.13] and the 0.5 mg/kg baricitinib group (FC = 13.97 ± 3.25) in comparison to the diabetic control, normal control and metformin treatment groups. There were no significant differences between the other treatment groups and the diabetic and normal control groups (Figure 5A).

#### 3.5.2 SIRT‐1

In the normal control group, liver SIRT‐1 gene expression was significantly increased (FC = 11.5 ± 0.96) compared to the diabetic control group. Similarly, coadministration treatment (FC = 11.6 ± 0.99) and baricitinib administered at 0.5 mg/kg and 2.5 mg/kg (FC = 8 ± 1.47 and 5.9 ± 0.84, respectively) also elevated liver SIRT‐1 gene expression levels. The combination treatment group showed a significant increase in liver SIRT‐1 gene expression compared to metformin (FC = 2 ± 0.27), 2.5 mg/kg and 5 mg/kg baricitinib (FC = 5.9 ± 0.84, 3.8 ± 0.57, respectively) (Figure 5B).

#### 3.5.3 NF‐ᴋB

The liver NF‐ᴋB gene expression levels did not significantly differ between the normal control group and the diabetic control group. However, compared to the diabetic control group, combination therapy (FC = 26.7 ± 2.1) and baricitinib at doses of 0.5 mg/kg (FC = 24.3 ± 4.2), 2.5 mg/kg (FC = 15.8 ± 1.18) and 5 mg/kg (FC = 16.9 ± 2.4) significantly increased liver NF‐ᴋB gene expression levels. Furthermore, NF‐ᴋB gene expression level in the metformin group (FC = 3.14 ± 0.6) was significantly reduced compared to combination treatment and baricitinib at doses of 0.5, 2.5 and 5 mg/kg (Figure 5C).

#### 3.5.4 SOCS1

A significant increase in liver SOCS1 mRNA expression was observed in the normal control group (FC = 14.8 ± 1.4) and combination treatment (FC = 15.9 ± 2.1) in comparison to the diabetic control group. In the combination treatment group, liver expression of SOCS1 showed a significant increase compared to the metformin group (1.76 ± 0.35), solvent group (FC = 6.5 ± 0.9) and baricitinib groups at doses of 0.5 mg/kg (FC = 2.97 ± 0.4), 2.5 mg/kg (FC = 3 ± 0.86) and 5 mg/kg (FC = 1 ± 0.4) (Figure 5D).

#### 3.5.5 SOCS3

Liver SOCS3 expression was significantly upregulated in the combination therapy group (FC = 11.3 ± 1.3) relative to the diabetic control group. There were no significant differences between the other treatment groups and the diabetic control group. Furthermore, liver SOCS3 mRNA expression was significantly increased in the combination treatment group compared to the metformin group (0.49 ± 0.08), and 0.5, 2.5 and 5 mg/kg baricitinib groups (0.23 ± 0.13, 1.31 ± 0.76, 0.5 ± 0.19, respectively) (Figure 5E).

## 4 Discussion

Clinical treatment strategies should target metaflammation, which is a key contributor to diabetes progression and its related complications. There is growing interest in combination drug therapy and the use of small molecule‐targeted drugs [25]. Metformin, widely prescribed for diabetes management [26], has well‐documented anti‐inflammatory properties [27]. On the other hand, baricitinib is prescribed for patients with rheumatoid arthritis who do not respond to conventional synthetic disease‐modifying antirheumatic drugs (cs DMARDs) [28]. Baricitinib is generally considered safe and well tolerated [29]. Evidence from several clinical studies involving patients with rheumatoid arthritis (RA) and T2DM suggests that baricitinib may exert a glucose‐lowering effect [30]. Therefore, here we assess the effects of the concomitant use of metformin and baricitinib as an anti‐inflammatory strategy in rats with T2DM.

We explored the effects of various doses of baricitinib on weight changes in diabetic rats. A previous study in mice demonstrated that treatment with tofacitinib (a JAK1 and JAK3 inhibitor) significantly increased the mitochondrial uncoupling protein 1 (UCP1) gene expression in white adipose tissue. By uncoupling oxidative phosphorylation from ATP production, UCP1 allows energy to be dissipated as heat rather than generated as ATP [31]. JAK inhibitor treatment is anticipated to reduce obesity, as our results demonstrated a dose‐dependent decrease in body weight with increasing doses of baricitinib. Consistently, an earlier study reported a significant reduction in body weight in mice fed high‐fat diets treated with 10 mg/kg of baricitinib [32]. However, Lian et al. observed no significant weight gain in diet‐induced obese C57BL/6J mice treated with 10 mg/kg of baricitinib for 12 weeks [33].

In the current study, metformin therapy administered at 150 mg/kg for 4 weeks did not significantly reduce blood glucose levels. These findings are supported by Majithiya et al., who reported similar results in diabetic rats using the same dose and treatment duration of metformin [34]. Additionally, Zhai et al. found that extending the treatment duration to 8 weeks at the same dose also failed to significantly reduce blood glucose levels. However, higher doses of metformin, such as 300 or 500 mg/kg, significantly reduced blood glucose concentrations [35]. This suggests that a dose exceeding 150 mg/kg is necessary to effectively reduce blood glucose levels in diabetic rats. However, higher doses were not employed in this study to allow for the evaluation of potential synergistic effects between baricitinib and metformin on blood glucose levels.

Impaired β‐cell function and insulin resistance are key contributors to diabetes mellitus. While insulin levels in our study did not significantly change, the HOMA‐IR index in all treatment groups reduced significantly compared to the diabetic control group. Notably, there was a significant increase in the HOMA‐B index in the group receiving 0.5 mg/kg baricitinib. Waibel et al. conducted a clinical trial on people with recent‐onset type 1 diabetes and evaluated the effects of baricitinib treatment over 1 year. They reported that baricitinib preserves β‐cell function for insulin production and reduces the daily insulin injections [36]. However, in our study, baricitinib did not significantly affect insulin production in the short term. Despite this, our results indicate that baricitinib exerts beneficial short‐term effects on HOMA‐IR after just 1 month of treatment. There is consistency between these findings and histopathological observations of the pancreas, which revealed improved pancreatic architecture, enhanced cell density and qualitative signs of regeneration across various therapy groups relative to the diabetic control group.

We demonstrated that 0.5 mg/kg of baricitinib treatment significantly increased LDL‐C, HDL‐C, TG and TC levels, and 2.5 mg/kg of baricitinib treatment significantly increased LDL‐C, TG and TC levels compared to the diabetic control group. A similar conclusion has been reached in trials involving patients with moderate‐to‐severe active RA, which reported that oral administration of baricitinib once daily was linked to elevated levels of TC, LDL‐C, HDL‐C and TG [37]. Similarly, another study in RA patients found that baricitinib treatment significantly increased HDL‐C, LDL‐C and TG levels [38]. A meta‐analysis study further supports these results, indicating that baricitinib causes a significant, dose‐dependent increase in HDL‐C and LDL‐C levels in RA patients [39]. Interestingly, the combination therapy of metformin and baricitinib counteracts the lipid profile alterations induced by baricitinib alone. This finding may have practical implications for patients incorporating baricitinib into their treatment regimen. Furthermore, photomicrographs of liver tissue revealed that various treatment groups alleviated pathological changes and improved hepatocyte structure and architecture compared to the diabetic control group.

We observed a significant decrease in serum TNF‐α levels in both the metformin and combination treatment groups compared to the diabetic control group. Although serum TNF‐α levels were increased in the diabetic control group in comparison to the normal control group, these alterations were not statistically meaningful. Consistently, researchers found no significant differences in serum TNF‐α levels among Brazilian patients with T2DM and non‐diabetic controls [40]. However, other studies in diabetic patients have documented elevated TNF‐α levels compared to non‐diabetic subjects [41, 42]. Conversely, Ahmed Al‐Shukaili et al. reported increased levels of IL‐10 and decreased levels of TNF‐α in patients with T2D in comparison with normal controls [43]. Additionally, studies have shown that NOD mice with STAT1 knockout (NOD/STAT1(−/−)) are protected from TNF‐α‐mediated beta‐cell death [44]. According to these results, TNF‐α contributes to the pathophysiology of beta cell loss and diabetes progression.

While direct evidence linking baricitinib to IL‐10 levels in humans or animals is limited, it is well established that baricitinib suppresses inflammatory pathways. Given its broad inhibitory effects on these pathways, baricitinib may increase IL‐10 levels, as an anti‐inflammatory cytokine. Consistent with this, our study found that combination therapy and various doses of baricitinib meaningfully elevated serum IL‐10 levels compared to the diabetic control group. Research also indicates that systemic overexpression of IL‐10 in NOD mice suppresses autoimmunity [45]. Furthermore, overexpression of IL‐10 in the muscle of transgenic mice protects against diet‐induced insulin resistance and inflammation [46]. However, local overexpression of IL‐10 in the pancreatic islets of NOD mice exacerbates diabetes and promotes insulitis [47]. Therefore, it seems that the role of IL‐10 in immune regulation and diabetes progression is tissue‐specific.

NF‐ᴋB signalling pathway stimulation by cytokines contributes to β‐cell dedifferentiation, impaired insulin secretion and ultimately β‐cell death [48]. Baricitinib may mitigate autoimmune‐mediated destruction or dysfunction of β cells by inhibiting NF‐ᴋB‐mediated inflammation, thereby preserving β‐cell function. In rats with haemorrhagic shock, baricitinib treatment reduced NF‐ᴋB activation and decreased the movement of p65 to the cell nucleus [49]. Studies on transgenic mice with conditional overexpression of IKK2 (an upstream activator of NF‐ᴋB) in β cells demonstrate that prolonged activation leads to the development of diabetes. Interestingly, when overexpression ceases, diabetes reverses and β cells regenerate [50]. Similarly, all treatment groups in our study showed a significant reduction in pancreatic NF‐ᴋB levels. These findings align with Prasath et al., who reported significantly elevated pancreatic NF‐ᴋB p65 levels in diabetic rats compared to controls [51]. However, liver NF‐ᴋB gene expression significantly increased in the combination and various baricitinib treatment groups. This suggests that its role in diabetes pathogenesis may have tissue‐specific regulatory mechanisms or differential drug targeting.

Notably, our study found no significant differences in serum levels of SIRT‐1 between the treatment groups, normal control group and the diabetic control group. While literature reporting serum SIRT‐1 levels remains limited, one study reported increased serum SIRT‐1 levels in normal control rats compared to diabetic rats [52]. Interestingly, liver SIRT‐1 gene expression was significantly elevated in the normal control group, combination group and the 0.5 and 2.5 mg/kg baricitinib groups. Consistently, transgenic mice overexpressing SIRT‐1 in pancreatic β cells exhibit improved glucose‐stimulated insulin secretion and increased glucose tolerance when fed with a high‐fat diet [53]. The observed increase in hepatic SIRT1 mRNA without corresponding changes in serum levels highlights two key points: first, the importance of tissue‐specific activity of SIRT‐1 because SIRT1 is predominantly nuclear/cytoplasmic in hepatocytes, with minimal secretion into circulation [54]. Increased hepatic mRNA may reflect localised adaptations that do not result in significant protein secretion into circulation. On the other hand, elevated mRNA does not necessarily translate to increased functional protein due to post‐translational regulation. Here, high glucose levels reduce cellular NAD^+^ concentrations. Since SIRT‐1 is an NAD^+^‐dependent enzyme, its activity may be diminished under these conditions [55]. Second, systematic detection limitations: Oxidative stress and systemic inflammation, both common in diabetes, may inhibit SIRT1 secretion or promote its degradation despite increased mRNA [56]. Together, these findings underscore the need to evaluate SIRT‐1 at tissue‐specific levels, as systemic measurements may not fully capture its metabolic regulatory roles.

**FIGURE 6:**
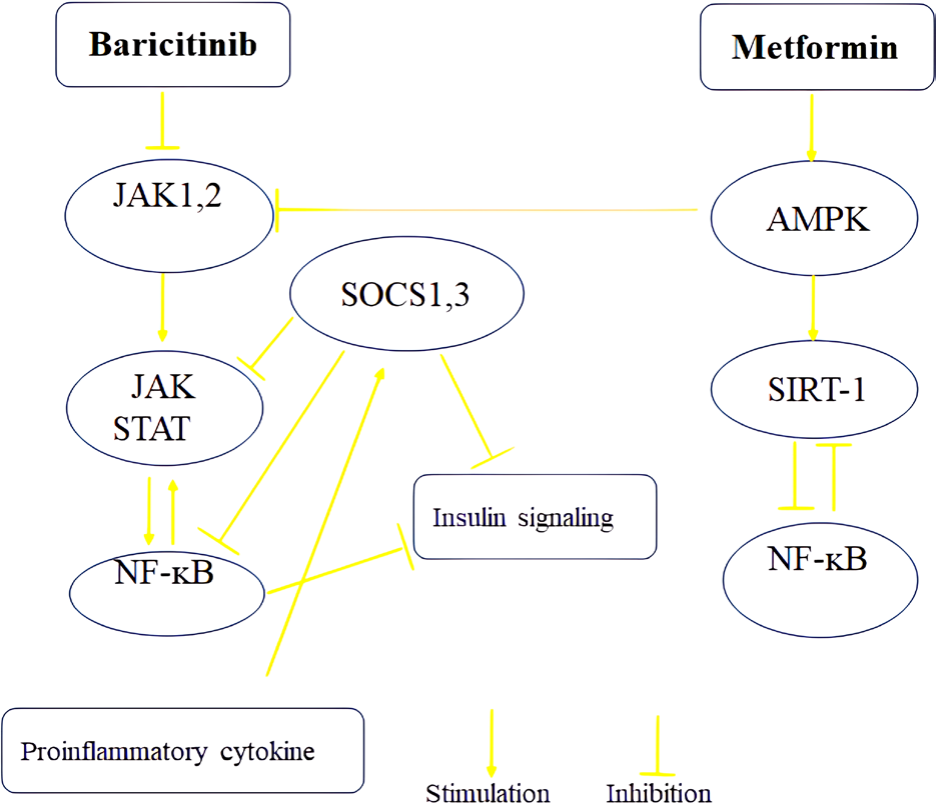
This schematic illustrates the potential interaction between metformin and baricitinib in mitigating metaflammation associated with diabetes. By combining these drugs, insulin sensitivity is enhanced, the production of anti‐inflammatory cytokines is promoted, and pro‐inflammatory cytokines are suppressed.

AMPK and SIRT‐1 mutually regulate each other and share numerous common molecular targets. Activation of the AMPK signalling pathway enhances SIRT‐1 gene expression, which subsequently inhibits NF‐ᴋB gene expression (Figure 6). This mechanism contributes to protection against obesity, inflammation and insulin resistance [8, 57]. Interestingly, our study revealed that combination treatment and 0.5 mg/kg baricitinib group exhibited increased liver gene expression of AMPK and SIRT1. Interestingly, 150 mg/kg metformin treatment alone did not alter the expression of these genes. As a result of these observations, it may be possible to activate overlapping protective pathways when metformin and baricitinib are taken together.

SIRT‐1 and NF‐ᴋB exhibit an antagonistic interaction in regulating inflammatory responses and metabolic disorders [58]. In our study, however, the combination group and all three doses of baricitinib significantly increased liver NF‐ᴋB gene expression compared with the diabetic control group, despite its pancreatic protein level being reduced. Consistently, liver SOCS1 or SOCS3 gene expression did not significantly alter in the baricitinib treatment groups. The increased hepatic mRNA levels likely reflect increased transcriptional demand in response to persistent inflammatory stimuli and oxidative stress in the diabetic liver microenvironment [59]. Baricitinib's inhibition of JAK–STAT signalling may further exacerbate this effect by disrupting SOCS1/SOCS3‐mediated negative feedback loops that normally suppress NF‐ᴋB activation [60].

On the other hand, the observed reduction in pancreatic NF‐ᴋB concentration could be attributed to two potential mechanisms: First, while NF‐ᴋB activation is crucial for mediating inflammatory responses and apoptosis in β cells during early diabetes [61], its subsequent decline may parallel progressive β‐cell loss. Second, as diabetes advances and β‐cell mass diminishes, decreased NF‐ᴋB protein levels may simply reflect reduced cellularity and impaired β‐cell function (Figure 6) [62].

A limitation of this study is that the effect of treatments was not assessed on antioxidant capacity in diabetic rat serum, liver and pancreas tissues. In addition, hepatic protein levels of AMPK, SIRT‐1, NF‐ᴋB, SOCS1, SOCS3 and β‐cell mass quantification (e.g., via Gomori's Aldehyde‐fuchsin Staining) were not investigated. These aspects need to be clarified in future studies to fully understand the treatments' mechanisms.

## 5 Conclusion

Our results demonstrate the weight‐reducing and insulin‐sensitising effects of baricitinib. For the first time, this study provides evidence that varying doses of baricitinib, particularly 0.5 mg/kg when combined with metformin, can reduce insulin resistance and improve the histopathological alterations of the liver and pancreatic islet cells in diabetic rats. These effects are mediated by the upregulation of hepatic AMPK and SIRT‐1 gene expression. Additionally, the combination therapy exhibits anti‐inflammatory properties, as evidenced by increased serum IL‐10 concentrations and reduced serum TNF‐α and pancreatic NF‐ᴋB concentrations. Furthermore, the combination therapy mitigated the adverse effects of baricitinib on the serum lipid profile. Based on these findings, baricitinib and metformin combination therapy appear to be a promising therapeutic strategy for diabetes mellitus. This warrants further investigation in future studies to confirm this.

## Author Contributions

A.J.M. supervised the study, contributed to the conceptualization of the research plan, designed the methodology and was involved in reviewing and editing the original draft. M.A. participated in the conceptualization of the research plan, handled the animal experiments, performed laboratory tests and contributed to writing and editing the original draft. M.S.J. provided expertise in molecular techniques and methodological advice. M.J. participated in the interpretation of pathological findings and the design of the methodology. In the final version of the manuscript, all authors have reviewed and approved it.

## Conflicts of Interest

A potential conflicts of interest has not been identified by the authors in this paper.

## Data Availability

The data that support the findings of this study are available on request from the corresponding author. The data are not publicly available due to privacy or ethical restrictions.
